# Not all autoantibodies are clinically relevant. Classic and novel autoantibodies in Sjögren’s syndrome: A critical review

**DOI:** 10.3389/fimmu.2022.1003054

**Published:** 2022-10-17

**Authors:** Francisco Vílchez-Oya, Hector Balastegui Martin, E. García-Martínez, Hèctor Corominas

**Affiliations:** ^1^Department of Anaesthesiology, Pain Medicine Section, Hospital Clínic de Barcelona, Barcelona, Spain; ^2^Department of Immunology, Hospital Universitario Gregorio Marañón, Madrid, Spain; ^3^Department of Rheumatology and Autoimmune Diseases, Hospital de la Santa Creu i Sant, Barcelona, Spain; ^4^Department of Medicine, Universitat Autònoma de Barcelona (UAB), Barcelona, Spain; ^5^Institut d'Investigació Biomèdica Sant Pau (IIB SANT PAU), Barcelona, Spain

**Keywords:** autoimmunity, Sjögren's syndrome, anti-Ro/SSA antibodies, Sjögren, antinuclear antibodies

## Abstract

Sjögren’s syndrome (SjS) is a heterogeneous systemic disease. The abnormal responses to La/SSB and Ro/SSA of both B-cells and T-cells are implicated as well as others, in the destruction of the epithelium of the exocrine glands, whose tissue characteristically shows a peri-epithelial lymphocytic infiltration that can vary from sicca syndrome to systemic disease and lymphoma. Despite the appearance of new autoantibodies, anti-Ro/SSA is still the only autoantibody included in the American College of Rheumatology/European League Against Rheumatism (ACR/EULAR) classification criteria and is used extensively as a traditional biomarker in clinical practice. The study and findings of new autoantibodies in SjS has risen in the previous decade, with a central role given to diagnosis and elucidating new aspects of SjS physiopathology, while raising the opportunity to establish clinical phenotypes with the goal of predicting long-term complications. In this paper, we critically review the classic and the novel autoantibodies in SjS, analyzing the methods employed for detection, the pathogenic role and the wide spectrum of clinical phenotypes.

## Introduction

Sjögren’s syndrome (SjS) is a systemic autoimmune disease which characteristically presents organ lymphocytic infiltration and has a specific predisposition for the exocrine glands. Its main consequence is the development of sicca symptoms, principally in the form of xerostomia and keratoconjunctivitis sicca (1). Nonetheless, a third of patients with long-standing SjS suffer from different systemic complications such as neurological, pulmonary or nephrological manifestations and nearly 5% of them end up developing lymphoma (2).

Multiple factors are involved in the pathogenesis of SjS and it is triggered in individuals with genetic predisposition by environmental factors. The core components of the disease process are autoimmunity and chronic inflammation secondary to the activation of an innate and adaptive immune response (1, 2).

It has been proven that the innate immune system plays a paramount role in early SjS through a type I interferon (IFN), cytokines such as IL-21 and the B-cell-activating factor (BAFF). The initial tissue and cell damage caused by different factors results in the production of IFN I and II in a first stage, inducing in turn the production of BAFF, which is responsible for the activation of autoreactive B-cells, among other processes. Thus, the TNF cytokine family connects innate immunity with the autoimmune activation of B-cells. Other cytokines, such as IL-12, are also central to its pathogenesis, by the activation of the type II IFN system through the innate immune system (natural killers’ cells), as well as the adaptive one *via* type 1 T-helper cells. In addition, many other molecules are also important in the pathogenesis, such as IL-21, with a role in the regulation of B-cells and follicular cells, and the CXCR5-CXCL13 axis, with a key role in lymphocyte recruitment and possibly, in the formation of ectopic germinal centers (GCs) (2).

B-cells up-regulation is a crucial feature of SjS, reflected by the broad array of autoantibodies found in the serum of those patients and confirmed by the presence of ectopic GCs in the affected organs tissue (2–4). The development of ectopic GCs on the site of inflammation, often within the salivary glands, has been associated with a higher frequency of local production of anti-Ro/SSA and anti-La/SSB autoantibodies in SjS patients (5). Ectopic GCs are functional structures that offer all the suitable machinery necessary for the activation of autoreactive B-cells and the production of autoantibodies. A complex array of cytokines and immune cells are thought to be involved in the formation of these structures. BAFF cytokine, a key molecule in SjS pathogenesis, is produced by infiltrated immune cells in salivary glands and regulates B-cells activation, proliferation and, importantly, B-cells selection through a ligand competition mechanism. Contrary to what happens in the bone marrow, BAFF has an effect in B-cells tolerance at the periphery, with increased levels of circulating BAFF leading to a decreased competition and resulting in the escape of autoreactive B-cells (6). Consistent with this finding, increased levels of BAFF in serum of SjS patients is correlated with the presence of anti-Ro/SSA and anti-La/SSB autoantibodies (7).

Serum autoantibodies are present in most patients with SjS, and some show a strong association with specific clinical features, possibly contributing directly to the phenotype of individual patients, though SjS classification criteria include just anti-Ro/SSA and anti-La/SSB (8, 9).

Recently, the research seeking novel autoantibodies in SjS has increased, opening up a wide range of clinical phenotypes in an attempt to predict long-term complications (8).

In this review, we will analyze different SjS autoantibodies, including old and newly identified biomarkers and discuss their utility as diagnostic tools, the pathogenic role and their association with some clinical phenotypes.

## Classic autoantibodies in Sjögren’s syndrome

### Anti-nuclear antibodies (ANA)

ANA are antibodies that target antigens in the nucleus of the cell. The gold standard method used for screening to detect ANA is the indirect immunofluorescence assay (IIFA) on HEp-2 cells, which is sensitive but not specific. The HEp-2 IIFA test provides further information than just whether or not autoantibodies are found, including antibody titration and the HEp-2 IIFA pattern (10). Higher antibody levels are associated with systemic autoimmune rheumatic diseases (SARD) and are more likely to identify a specific autoantigen during patient follow-up (10–12). It is of importance that ANAs are widespread in the healthy population and are not suitable for screening in connective tissue diseases, thus a positive result for a specific ANA in the absence of a clinical context or clinical findings is not equivalent to the diagnosis of a connective tissue disease.

To summarize the classification and descriptions of the specific patterns of HEp-2 IIFA, the international consensus on ANA patterns (ICAP) established each pattern and sub-pattern, which were identified with an anti-cell (AC) pattern code (AC-1 to AC-29) (10, 13). The HEp-2 IIFA antibody patterns, their specific antigens and their clinical relevance in SARD are summarized in **Table 1**.

**Table 1:** HEp-2 cell IIFA patterns and their correlated clinical relevance in Systemic Autoimmune Rheumatic Diseases (SARD) (10, 13).

Code	AC pattern	Specific antigens	Clinical relevance
Nuclear patterns			
AC-1	Homogeneous	Nucleosome (dsDNA, ssDNA, histone)	SLE, chronic autoimmune hepatitis, juvenile idiopathic arthritis.
AC-2	Dense fine speckled	DSF70/LEDGF	Healthy individuals or patients without systemic autoimmune rheumatic diseases (SARD).
AC-3	Centromere	CENP-A/B	Limited cutaneous SSc.
AC-4	Fine speckled	SS-A/B, Mi-2, TIF1gamma, TIF1betta, Ku	SjS, SLE, subacute cutaneous lupus erythematosus, neonatal lupus erythematosus, congenital heart block, DM, SSc and SSc-AIM overlap syndrome.
AC-5	Large/coarse speckled	Sm, U1RNP, RNA polymerase III	SLE, SSc, MCTD, SSc-AIM overlap syndrome, UCTD.
AC-6	Multiple nuclear dots	Sp-100, PML proteins, MJ/NXP-2	PBC, AIM (DM) and other inflammatory conditions.
AC-7	Few nuclear dots	P80-coilin, SMN	Low positive predictive value for any disease.
AC-8	Homogeneous nuclear	PM/Scl-75, PM/Scl-100, Th/To. B23/nucleophosmin, nucleolin, No55/SC65	SSc, SSc-AIM overlap syndrome.
AC-9	Clumpy nucleolar	U3-RNP/fibrilarin	SSc.
AC-10	Punctate nucleolar	RNA polymerase I, NOR-90	SSc, Raynaud’s phenomenon, SjS and cancer.
AC-11	Smooth nuclear envelope	Lamins A, B, C lamin-associated proteins	Autoimmune cytopenias, autoimmune liver diseases, linear scleroderma, APS and other SARDs.
AC-12	Punctate nuclear envelope	Nuclear pore complex proteins (gp210, p62, LBR, Tpr)	PBC, autoimmune liver diseases.
AC-13	PCNA-like	PCNA	SLE (debated).
AC-14	CENP-F like	CENP-F	Neoplastic conditions (breast, lung, colon, lymphoma, ovary, brain).
AC-29	TOPOI-like	DNA-topoisomerase/SCL70	SSc (particularly with diffuse cutaneous SSc and more aggressive forms).
**Cytoplasmatic patterns**			
AC-15	Fibrillar linear	F-actin, non-muscle myosin	AIH type 1, chronic HVC infection, celiac disease. Rare in SARD.
AC-16	Fibrillar filamentous	Cytokeratin, vimentin, tropomyosin	Not typically found in SARD.
AC-17	Fibrillar segmental	Alpha-actinin, vinculin	SLE, AIH type I, chronic inflammatory demyelinating neuropathy.
AC-18	Discrete dots	GW (182)	Neurological symptoms in a variety of diseases.
AC-19	Dense fine speckled	PL-7, PL-12, ribosomal P proteins	SLE, anti-synthetase syndrome, interstitial lung disease, polyarthritis, Raynaud’s phenomenon, mechanic’s hands.
AC-20	Fine speckled	Jo-1	Anti-synthetase syndrome, interstitial lung disease, polyarthritis, mechanic’s hands.
AC-21	Reticular/AMA	Mitochondrial structures: PDC-E2/M2, BCOADC-E2, E1alpha, E3BP/protein X	PBC, SSc, PBC-SSc overlap syndrome and PBC-SjS overlap syndrome.
AC-22	Polar/Goldi-like	Golgi structures: giantin/macrogolgin, golgin-95/GM130, golgin-160, golgin-97, golgin-245	Small number of patients with: SjS, SLE, RA, MCTD, GPA, adult onset Still’s disease, viral infections.
AC-23	Rods and rings	IMPDH2	HCV patients treated with pegylated interferon-alpha/ribavirin combination therapy.
**Code**	**AC pattern**	**Specific antigens**	**Clinical relevance**
**Mitotic patterns**			
AC-24	Centrosome	Pericentrin, ninein, Cep250, Cep110	Low positive predictive value for any disease.Low percentage in SSc, SLE, Raynaud’s phenomenon.
AC-25	Spindle fibers	HsEg5	Low positive predictive value for any disease.SjS and SLE (not specific).
AC-26	NuMA-like	NuMA	SjS, SLE, UCTD, limited SSc, RA.
AC-27	Intercellular bridge	CENP-E, CENP-F, TD60, MSA36, KIF-14, MKLP-1, MPP1/KIF20B.	Low positive predictive value for any disease.Very rare: SSc, SLE, Raynaud’s phenomenon and malignancy.
AC-28	Mitotic chromosomal	DNA-topoisomerase/SCL70	SSc (difuse).

AIM, autoimmune myopathy; AMAs, antimitochondrial antibodies; APS, antiphospholipid syndrome; CENP, centromere-associated protein; DFS, dense fine speckled; DM, dermatomyositis; ENA, extractable nuclear antigens; HCV, hepatitis C virus; IIFA, indirect immunofluorescence assay; LAP, lamin-associated polypeptide; LBR, lamin B receptor; LEDGF, lens epithelial derived growth factor; NOR, nucleolus organiser region; NXP, nuclear matrix protein; PBC, primary biliary cholangitis; PCNA, proliferating cell nuclear antigen; PML, promyelocytic leukaemia; PM/Scl, polymyositis-scleroderma; RA, rheumatoid arthritis; RNApol, RNA polymerase; RNP, ribonucleoprotein; SARD, systemic autoimmune rheumatic diseases; SLE, systemic lupus erythematosus; SMN, survival of motor neuron; SSc, systemic sclerosis; SjS, Sjoügren’s syndrome; TIF, transcription intermediary factor; TRIM, tripartite motif; Tpr, translocated promoter region; UCTD, undifferentiated connective tissue disease; ssDNA, single stranded DNA; dsDNA, double stranded DNA; hUBF, human upstream binding factor; AIH, autoimmune hepatitis; Ago, argonaute protein; CLIP, class II-associated invariant chain peptide; EEA, early endosome antigen; SRP, signal recognition protein; tRNA, transfer ribonucleic acid; Cep, centrosomal protein; DCA, dividing cell antigen; INCENP, inner centromere protein; KIF, kinesin family; MCA, mitotic chromosomal antigen; MKLP, mitotic kinesin-like protein; MPP, M-phase phosphoprotein; MSA, mitotic spindle apparatus; NMP, nuclear matrix protein; NuMA, nuclear mitotic apparatus; PCM, pericentriolar material; UCTD, undifferentiated connective tissue disease.

ANA have been found to be positive in 59 to 85% of SjS patients in whom a higher prevalence of recurring parotidomegaly and a heightened frequency of extra-glandular symptoms are observed (14, 15).

**Figure 1:**
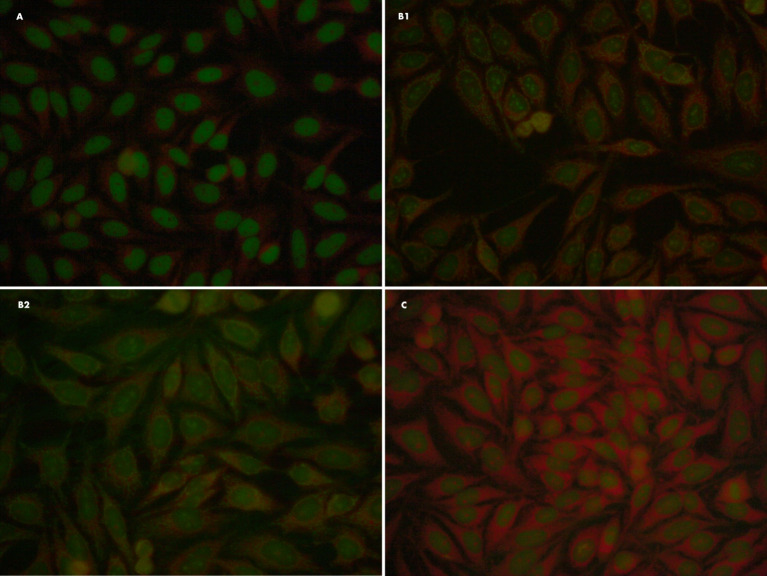
Different ANA patterns of primary SjS patients. **(A)** ANA IFI: Fine speckled (AC-4) 1/160. Negative specificity study for extractable nuclear antigens (ENAs). **(B1, B2)** ANA IFI: nuclear dots/nucleolar pattern (AC-7/10) 1/80; it also presents a minor reticular cytoplasmic pattern (AC-21) compatible with anti-mitochondrial antibodies. Organ-specific antibodies: specifity M2/nPCD. **(C)** Patient with anti-Ro/SSA antibody (Ro-52 kDa) and negative ANA-IFI on HEP2-cells.

The naming of the autoantigens is based on their biochemical structure or the associated disease, and the antigens in Sjögren’s syndrome are named SS-A/B. The frequency of elevated ANA titres in SjS is 40-95%. In suspected SjS patients, one of the most characteristic patterns is AC-4 (nuclear fine speckled), in which case it would be advisable to have follow-up tests done in search of anti-Ro/SSA and anti-La/SSB antibodies (10). **Figure 1** shows different ANA patterns of primary SjS patients.

### Anti-Ro/SSA

Throughout many autoimmune diseases, anti-Ro/SSA antibodies appear to be the most prevalent specificity. Specially among patients with SjS and in the lupus spectrum (SLE, cutaneous lupus, congenital heart block and neonatal lupus). The Ro antigen consists of two proteins that weight 52 and 60 kDa. In humans, Ro 60 kDa is a ribonucleoprotein which forms small cytoplasmic complexes with non-coding RNAs (known as Y RNA). Among its functions, it mediates the quality control of misfolded non-coding RNAs and is involved in diverse cellular-stress responses such as survival after ultraviolet radiation damage (16). Ro 52-kDa (or TRIM21) is a E3 ubiquitin ligase implicated in the ubiquitination of many inflammatory related proteins like IRF transcription factors associated to IFN-I pathway. In addition, acts as cytosolic Fc receptor, being able to bind diverse isotype antibodies and playing a role in intracellular antibody-mediated immunity (17, 18). The human 60 kDa Ro protein is encoded by a 1.8 kb gene in chromosome 19 which possesses an RNA binding site, whereas the gene responsible for the 52 kDa Ro fraction is located in chromosome 11 and lacks any specific RNA binding domain (19, 20). Although RNA precipitation assay has showed the highest specificity and sensitivity to detect anti-Ro/SSA as well as anti-La/SSB, it can’t be used in routine analysis, in spite of its usefulness as a reference and confirmation assay (19, 20). Therefore, counter-immunoelectrophoresis (CIE) is regarded as the most specific (100%) and sensitive (89%) assay in order to find anti-Ro/SSA antibodies, and better than enzyme-linked immunosorbent assays (ELISA) or the Immunoblotting (IB) assay (19, 21).

ELISA is frequently used in order to detect these antibodies, since they are straightforward to perform and the interpretation of the results is fast. However, ELISA is not more specific than other kind of analyses. Depending on the commercial kit, various types of antigens, such as recombinant, native proteins and synthetic peptide fragments are used for the detection of SSA/SSB antibodies. Nevertheless, their sensitivity and specificity may vary notably, from 39 to 77% and from 79 to 100% respectively, and most of the ELISAs detecting anti-Ro/SSA and anti-La/SSB antibodies are generally specific for a single target, in spite of a few assays being able to detect several antibodies (22). The Immunoblotting (IB) assay showed high specificity for anti-60 and anti-52 kDa (97 and 95% respectively), but lower overall sensitivity (17 and 36% respectively) (19, 20). Meanwhile, line immunoassay (LIA), an enzyme-linked immunosorbent assay, showed a much better sensitivity (91.4%) and specificity (87%) for detecting anti-Ro/SSA (19, 23).

In clinical practice, anti-Ro/SSA antibodies can be associated with a wide range of SARDs, such as subacute cutaneous lupus, neonatal lupus, systemic lupus erythematous (SLE), SjS, SjS/SLE overlap syndrome, myositis, rheumatoid arthritis (RA) and primary biliary cirrhosis (24–28). Anti-La/SSB and anti-52 Ro antibodies are both directly implicated in the pathogenesis of neonatal lupus. In this context, fetal complete heart block is due to tissue injury mediated by the expression of Ro (both 52 and 62 kDa fractions) and La antigens in the cardiac tissue, particularly located on the surface of myocardial cells’, from the 18^th^ to the 24^th^ week. Although the main autoantibody involved in the neonatal pathogenesis is anti-Ro 52 (19, 29).

In SjS, anti-Ro/SSA and anti-La/SSB antibodies have been associated with diagnoses at a younger age, recurring parotidomegaly, more severe dysfunction of the exocrine glands, a more intense lymphocytic infiltration of the minor salivary glands and longer disease duration (30, 31). In addition, some authors suggest a higher prevalence of extra-glandular manifestations, for instance Raynaud’s phenomenon, vasculitis, splenomegaly and lymphadenopathy (30, 32).

### Anti-La/SSB

The La/SSB antigen is a phosphorylated protein which weighs 48 kDa. It can be found in the nucleus and the cytoplasm and binds to several RNA molecules (19). It participates in a broad spectrum of RNA metabolism processes as protecting nascent RNA polymerase III transcripts from exonuclease digestion by binding to their poly(U) termini, processing of 5’ and 3’ ends of pre-tRNA precursors and resolving misfolded RNA structures by acting as RNA chaperone (33). IgG anti-La/SSB antibodies are the major antibody class found in serum and it has been observed its strong association with anti-Ro/SSA antibodies. The latter can be found on their own in many patients’ sera, whereas anti-La/SSB are generally found together with anti-Ro/SSA. The presence of anti-La/SSB without anti-Ro/SSA antibodies hasn’t got any significant association with SjS phenotypic characteristics (34).

Anti-Ro/SSA and anti-La/SSB antibodies are commonly detected in patients suffering from cryoglobulinemia, hypergammaglobulinemia and in the presence of rheumatoid factor independently of autoimmune disease (19, 35).

## Autoantibodies in Sjögren’s syndrome associated with overlap syndromes

### Anti-mitochondrial antibodies (AMA)

AMAs are the serologic distinguishing finding of primary biliary cholangitis (PBC). AMAs are found in 95% of patients with PBC with a specificity of 98% for the disease (36).

There are four main autoantigens targeting antimitochondrial antibodies, referred to as an M2 subtype of mitochondrial autoantigens and which are identified as the E2 subunits of the pyruvate dehydrogenase complex, the ketoglutaric acid dehydrogenase complex, the branched chain 2-oxo-acid dehydrogenase complex and the dihydrolipoaminade dehydrogenase-binding protein (37). When IIFA is used, between 1.7 and 13% of SjS patients test positive for AMA. However, the positivity rate is higher (3 to 27%) if Western Blot or ELISA is used (38–40). Some SjS patients with a positive AMA test, besides developing more frequently hepatic manifestations, show also a higher prevalence of hypergammaglobulinaemia, peripheral neuropathy, Raynaud’s phenomenon and higher titer in the erythrocyte sedimentation rate (ESR) (14, 38).

The similarity of the histological lesion in PBC and SjS is noteworthy. Both are characterized by lymphocytic infiltrates with a predominance of CD4^+^ cells that initiate around the ductal epithelium (salivary or bile ducts). Hence, both entities may share some pathogenetic mechanisms within the context of some kind of autoimmune epithelitis, in spite of their different autoantibody profiles (38, 41).

### Anti-centromere antibodies (ACA)

ACAs are a heterogeneous group of antibodies targeting different antigens clustered around the kinetochore plates (42). In systemic sclerosis (SSc), ACAs recognize three centromeric proteins (CENP): CENP-A (19 KDa), CENP-B (89 KDa) and CENP-C (140 KDa). Anti-CENP-B autoantibodies seem to have a greatest relevance for clinical practice among the various ACA, with a sensitivity of 20 to 30% for SSc (42). Among SARD, ACAs are also present in approximately 15% of patients with PBC and this association has been described with worsening outcomes compared in those seronegative patients (43).

In SjS, the prevalence of ACAs ranges from 3.7 to 27% when IIFA is used to detect them, but when ACAs are detected by other methods its prevalence varies between 20 and 27%, depending on which CENP is used as target antigen (44–46). CENP recognition patterns in SjS and SSc are dissimilar. Most SjS sera recognize CENP-C alone (70% of SjS versus 6% of SSc), whereas most SSc sera recognize CENP-B as well as CENP-C (83% of SSc versus 0% of SjS) (38, 46).

Regarding immunological and clinical features, some studies have shown that seropositive ACA patients with SjS have a higher disease onset mean age, a higher frequency of Raynaud’s phenomenon, peripheral neuropathy and keratoconjunctivitis sicca compared to seronegative patients (38, 47, 48). Moreover, ACA positive SjS patients showed an inferior prevalence of anti-Ro/SSA and anti-La/SSB antibodies, a lower frequency of cytopenia, inferior rates of rheumatoid factor and hypergammaglobulinaemia (38, 49).

The singular features presented in ACA positive SjS patients suggests that they could be a SjS clinical subset with some overlapping traits of SjS and SSc (38). Interestingly, a study of SjS patients by capillaroscopy showed that those patients with ACA had a scleroderma-like pattern in 10.2%, showing dilated capillaries, low capillary density and pericapillary haemorrhages, raising the suspicion of an overlapping syndrome between SjS and SSc (50). The percentage of patients progressing to SSc span from 0 to 40% according to different cohorts (38, 49, 51), even though more accurate and prospective design studies are needed to determine it.

### Anti-citrullinated peptide antibodies (ACPAs)

ACPAs are autoantibodies targeting proteins that have been citrullinated by a calcium-dependent enzyme family of peptidylarginine deiminases. Its specificity in RA is around 95% and it identifies a phenotype of patients affected by a more serious clinical profile and erosive disease (52). It is widely known that SjS may exist together with other autoimmune diseases, being autoimmune hypothyroidism the most frequent, followed by RA (53). In patients with SjS, the prevalence of ACPAs ranges between 3 and 9.9%, but no agreement exists regarding its clinical significance (54–58), although a small number of authors have described an association with non-erosive arthritis (54, 55, 57).

Some reports have described a low progression to RA in cohorts of patients with SjS during a long-term follow-up (59, 60). Nonetheless, it has been suggested that ACPAs positive SjS patients may have a higher risk of progression to RA (57, 61). Thus, ACPAs constitute a valuable tool for the accurate control of this subgroup of patients in order to ensure their immediate diagnosis and treatment (62).

## Novel autoantibodies in Sjögren’s syndrome

As previously commented, classical autoantibodies have been used extensively in the setting of SjS diagnosis and currently, anti-Ro/SSA is widely accepted and considered useful for SjS classification criteria as a diagnostic tool (9).

In recent years, several studies have analyzed new autoantibodies, opening up a window of opportunities for the diagnostic approach, trying to discern the pathologic attributions of disease-associated autoantibodies and providing the opportunity to identify pre-clinical subjects before SjS onset (8).

Despite the fact that most of these antibodies are infrequent and, may even be shared with other connective tissue diseases, their study may help to elucidate some of the clinical features of patients with SjS and help to tackle potentially serious complications in those patients with suspected or confirmed SjS, as well as proving to be useful in order to reach a diagnosis when other autoantibodies turn out to be negative. Among the antibodies that we will discuss are anti-alphafodrin, anti-muscarinic type 3 receptor (M3R), anti-salivary gland protein 1 (SP1), anti-carbonic anhydrase, anti-parotid secretory protein (PSP), anti-interferon-inducible protein-16, anti-NA-14, anti-MDM2, anti-stathmin-4, anti-PUF60, anti-NR2, anti-TRIM38, anti-calponin-3, anti-saccharomyces cerevisiae (ASCA), anti-aquaporin (AQ), anti-ganglionic acetylcholine receptor (gAChR), anti-P-selectin, anti-moesin, anti-carbamylated, anti-alpha-enolase, anti-cofilin-1 and anti-Rho GDP-dissociation inhibitor 2 (RGI2) (63–92).

In addition, it is worth highlighting two ANA patterns mentioned in **Table 1** and observed in patients with SjS that may have additional clinical value in the spectrum of clinical manifestations. These are the antibodies for nuclear transcription factor NOR 90/hUBF (anti-NOR 90) and anti-NuMA antibodies. Anti-NOR 90, are antinuclear antibodies that recognize the distal ends of the short arms of chromosomes 13, 14, 15, 21 and 22 (nuclear organizer region) on immunofluorescence analysis and 90-kd doublet proteins on immunoblot analysis (93). Fujii T et al. identified that anti-NOR 90 antibodies are rare, although they are associated with SjS and overlap syndromes (SjS-RA and SjS-SSc) in Japanese patients. Anti-NuMA antibodies (antinuclear mitotic apparatus), although rare, are mostly associated with SLE and SjS, and could be useful in order to reach a diagnosis when other autoantibodies are negative. Those patients’ both clinical and biological profiles were milder, suggesting that these antibodies may imply a good prognosis marker in both syndromes. SjS patients with anti-NuMA antibodies presented fewer ocular sicca syndrome and dryness complications and anti-Ro/SSA and/or anti-La/SSB antibodies were less frequently present. Anti-NuMA positive patients received antimalarial agents less frequently than negative patients, with no difference regarding other treatments (94).

In **Table 2**, we summarize the novel autoantibodies found in SjS patients that may be associated with some clinical features.

**Table 2:** Novel autoantibodies in Sjögren’s syndrome.

Author	Number of patients	Autoantibodies	Technique	Prevalence	Sensitivity and specificity	Clinical features
Hu Q at al. (63)	Meta-analysis	**Anti-alpha fodrin**	Immunoblot and ELISA	38-42%	Sensitivity 39.3%Specificity 83%	Moderate accuracy for the diagnosis of SjS.Clinical manifestations were not evaluated.
Willeke P et al. (64)	62		ELISA	31-35%	Sensitivity 31-35%Specificity unknown	Shorter disease duration. Increased prevalence of recurrent parotid swelling with IgG isotype.
Mona M et al. (65)	156	**Anti-muscarinic type 3 receptor (M3R)**	On-Cell-Western assay	N/A	Sensitivity 75-98%Specificity 85%	Correlated with ocular dryness and glandular hypofunction and the haematological/biological domains of ESSDAI. Useful in SjS diagnosis, especially where clinical assessments are limited.
Deng C et al. (66)	956		ELISA	N/A	Sensitivity 4-98%Specificity 58-100%	Potential diagnostic biomarker for SjS.Clinical features were not evaluated.
Shen L et al. (67)	123	**Anti-salivary gland protein 1 (SP1)**	ELISA	19% isolated34% associated to other autoantibodies.	N/A	Associated with anti-Ro/SSA and anti-La/SSB. No distinct clinical manifestations were identified in patients expressing anti-SP1.
Xuan J et al. (68)	134		Western-blot	40%	N/A	Higher levels during earlier stages of the disease.
Karakus S et al. (69)	136		N/A	27 %	N/A	Dry eye. Correlated with having a Schirmer test ≤ 5 mm.
Karakus S et al. (69)	136	**Anti-carbonic anhydrase 6 (CA6) and anti-parotid secretory protein (PSP)**	N/A	27% (CA6)54% (PSP)	N/A	Anti-CA6 was associated with severe ocular surface staining (corneal and conjunctival). Anti-CA6 may indicate early stages of SjS.Anti-PSP was the only autoantibody that correlated with primary SjS.
Pertovaara M et al. (70)	74	**Carbonic anhydrase auto-antibodies (CA)**	ELISA	N/A	N/A	CA-II, CA-VI and CA XIII (associated with renal manifestations).CA-VII and CA-XIII (correlated to β_2_ microglobulin)CA-I (oral dryness and associated with interstitial lung disease in other connective tissue diseases).
Alunno A et al. (71)	30	**Anti-Interferon-Inducible Protein-16**	ELISA	33%	N/A	Pathogenesis of glandular inflammation.
Baer AN et al. (72)	133		ELISA	29%	N/A	Severe disease: greater prevalence of abnormal Schirmer’s test, ANA >1:320 and germinal center-like structures in the labial salivary gland lymphocytic infiltrates. Focus scores were significantly higher.
Alunno A et al. (73)	67		ELISA	34%	N/A	Pathogenesis of glandular inflammation.
Uomori K et al. (74)	72	**Anti-NA-14**	ELISA	11.1%	N/A	Elevation of IgA levels. Low prevalence of ANA positive patients. Disease duration tended to be shorter (although the difference did not reach statistical significance).
Liu Y et al. (75)	100	**Anti-MDM2**	ELISA	21%	N/A	Longer disease duration and more lymphocytes focal gathering in labial gland. Higher prevalence of anemia, thrombocytopenia and anti-Ro/SSA.
Duda S et al. (76)	72	**Anti-stathmin-4**	ELISA	33% (pSS with PNP)7.8% (pSS without PNP)15% in sSS	N/A	Polyneuropathy.
Zhang YM et al. (77)	79	**Anti-PUF60**	ELISA and immunoblotting	10.1%	N/A	Overlap syndrome with myositis.
Fiorentino DF et al. (78)	84		ELISA	30%	Specificity 29%	May be more associated with Asian and African-American ethnicity, hypergammaglobulinemia, anti-Ro/SSA, anti-La/SSB and rheumatoid factor.
Lauvsnes MB et al. (79)	66	**Anti-NR2**	ELISA in serum and electrochemiluminescence in CSF	20%	N/A	Cognitive disturbances and mood disorders.
Lauvsnes MB et al. (80)	50		Electrochemiluminescence in CSF	12%	N/A	Loss of hippocampal gray matter.
Tjensvoll AB et al. (81)	71		Electrochemiluminescence in CSF	N/A	N/A	Cognitive impairment.
Wolska N et al. (82)	235	**Anti-TRIM38**	TNT Quick coupled transcription/translation system and immunoprecipitation assay	10.21%	N/A	Higher severity of disease: severe sialadenitis, higher van Bijsterved scores and lower Schirmer’s test scores.
Birbaum J et al. (83)	209	**Anti-calponin-3**	ELISA	11%	N/A	Neuropathy.
Alunno A et al. (84)	104	**Anti-saccharomyces cerevisiae (ASCA)**	Immunodot test	4.8%	Very low sensitivity, 100% specificity	Patients displayed a triple combination of circulating anti-Ro60/SSA, anti-Ro/52/SSA and anti-La/SSB antibodies associated with low complement and cutaneous involvement.
Birnbaum J et al. (85)	109	**Anti-aquaporin (AQ)**	Fluorescence-activated cell sorting (FACS) assay	10%	N/A	Neuromyelitis optica spectrum disorder.
Alam J et al. (86)	112		Indirect immunofluorescence assay	76.8%	Sensitivity 73%Specificity 68%	Low resting salivary flow.
Tzartos JS et al. (87)	34		ELISA verified by radioimmunoassay, western blot and AQP-transfected cells.	38.2%	N/A	Severe xeropthalmia, suggesting a potential pathogenic role.
Mukaino et al. (88)	39	**Anti-ganglionic acetylcholine receptor (gAChR)**	LIPS assay	23.1%	N/A	Autonomic symptoms.
Hu YH et al. (89)	70	**Anti-P-selectin**	ELISA	40.6% (SjS patients with thrombocytopenia) and 7.8% (SjS patients without thrombocytopenia).	N/A	May lead to platelet destruction and endothelial injury. Possible role in the pathogenesis of thrombocytopenia.
Zhang Y et al. (90)	50	**Anti-moesin**	ELISA	42%	N/A	N/A
Bergum B et al. (91)	78	**Anti-carbamylated**	ELISA	27%	N/A	Increased focal lymphocytic infiltration, formation of ectopic GC-like structures in minor salivary glands and diminished salivary gland function.
Cui L et al. (92)	70	**Anti-cofilin-1**	ELISA in saliva samples	N/A	Sensitivity 80%Specificity 90%	May predict progression to MALT lymphoma
Cui L. et al. (92)	70	**Anti-alpha-enolase**	ELISA in saliva samples	N/A	Sensitivity 90%Specificity 84%	May predict progression to MALT lymphoma
Cui L. et al. (92)	70	**Anti-Rho GDP-dissociation inhibitor 2 (RGI2)**	ELISA in saliva samples	N/A	Sensitivity 90%Specificity 80%	May predict progression to MALT lymphoma

ELISA, enzyme-linked immunosorbent assay; SjS, Sjögren’s Syndrome; ESSDAI, EULAR Sjögren’s syndrome disease activity index; ANA, antinuclear antibodies; anti-NA-14, nuclear autoantigen of 14 KDa; anti-MDM2, human homologue of mouse double minute 2; pSS, primary Sjögren ‘s Syndrome; sSS, secondary Sjögren’s Syndrome; anti-PUF60, poly(U)-binding-splicing factor 60 KDa; anti-NR2, N-methyl-D-aspartic acid receptor 2; CSF, cerebrospinal fluid; anti-TRIM38, tripartite motif-containing protein 38; LIPS assay, luciferase immunoprecipitation system assay; MALT, mucosa-associated lymphoid tissue; N/A, not available.

## Other relevant antibodies: Rheumatoid factors and cryoglobulins

Rheumatoid Factors (RF) and cryoglobulins, require special attention for their important clinical implications in SjS, partly due to their relationship with potentially serious disease complications.

### Rheumatoid Factors (RF)

Rheumatoid factors (RF) are autoantibodies targeting the constant part (Fc) portion of other immunoglobulins. The most distinguished are IgM and IgA binding to the Fc portion of IgG (95).

RF levels are found in 36 to 74% of SjS patients with an increased prevalence during follow-up and have been unmistakably associated with the number of extra glandular features (14, 32). The wide spectrum of clinical manifestations in patients with RF includes a higher frequency of articular damage, Raynaud’s phenomenon, parotidomegaly, cutaneous vasculitis, cytopenias, renal involvement and central nervous system manifestations (32, 96). In addition, RF levels are correlated to an active serological profile with higher presence of some antibodies, including ANA, anti-Ro/SSA, anti-La/SSB and cryoglobulins, as well as hypergammaglobulinaemia and hypocomplementemia (14, 32, 38). RF have been associated with diagnoses at a younger age and with lymphocytic infiltration in the biopsy of salivary glands. This last is associated with the lymphomagenesis process related to the role of monoclonal RF secreted by the B-cells through the chronic stimulation at the target organ (95).

### Cryoglobulins

Cryoglobulinemia is characterized by the precipitation of circulating immunoglobulins at temperatures down 37°C. Serologically, it is classified into three subtypes according to the composition of the immunocomplex. Thus, it is differentiated as type I cryoglobulinemia (monoclonal immunoglobulins), type II (mixed cryoglobulinemia containing monoclonal and polyclonal immunoglobulins) and type III (mixed cryoglobulinemia containing only polyclonal immunoglobulins). Type II cryoglobulinemia is commonly associated with infectious diseases, with special mention to hepatotropic viruses, while type III cryoglobulinemia is most commonly associated with autoimmune connective tissue diseases (95).

Cryoglobulins differentiate a subgroup of SjS patients with a worse prognosis, as they have been associated with an increased risk of lymphoproliferative disease (96). The wide spectrum of clinical manifestations in patients with cryoglobulinemia includes a higher frequency of Raynaud’s phenomenon, parotidomegaly, vasculitis, cytopenias, renal involvement and peripheral neuropathy (32, 96). Cryoglobulinemia is also commonly associated with the presence of other immunological markers such as hypocomplementemia, RF and anti-Ro/SSA (32, 96).

## Discussion

The pathogenesis of SjS is still elusive, although the role of abnormal autoreactive B-cells, which lead to the production of autoantibodies and the formation of immune complexes, seems to be fundamental in developing the syndrome. The presence of these autoantibodies is one of the hallmarks of SjS. Amid them, the most common are antibodies against Ro/SSA and La/SSB ribonucleoprotein complexes, which are included in the classification criteria of the American College of Rheumatology/European League Against Rheumatism (ACR/EULAR). Both anti-Ro/SSA and anti-La/SSB positive SjS patients are diagnosed at a younger age, have a graver glandular dysfunction and a higher frequency of extraglandular features.

One of the diagnostic criteria for the majority of autoimmune diseases is the presence of autoantibodies, whose detection may be overlapping in the spectrum of other autoimmune diseases. As commented throughout this review, there are no specific autoantibodies for SjS. Anti-Ro/SSA and anti-La/SSB constitute a diagnostic marker for the disease, however, these autoantibodies are found in up to 30-40% and 10-15% of patients with SLE, respectively, but anti-Ro/SSA and anti-La/SSB autoantibodies are not the only ones that can be linked to SjS or SLE. To data, these autoantibodies do not correlate with the etiopathogenesis of the disease, although in pregnancy, it may increase the risk of developing neonatal lupus (97). The detection of ANAs has been considered one of the most sensitive techniques in the screening of autoimmune diseases, but may appear related to other conditions such as infectious diseases, malignancy or in healthy population. There are numerous shared autoantigens between SjS and SLE, and it seems that the differential pathogenic mechanism between them may be the inflammatory tropism at the glands in primary Sjögren’s Syndrome, although the molecular explanation of that is still unknown (2). For all these reasons, despite that autoantibodies are important tools in the diagnosis of autoimmune diseases, they may be analyzed in a specific clinical context to evaluate their diagnostic value.

Antinuclear antibodies are serological biomarkers with a crucial role in the classification of systemic autoimmune rheumatic diseases. There is a continuous needed for harmonization of the methods for autoantibody determinations and in the research setting for the identification of novel autoantibodies, where several assay methods have become available in the last decades.

The HEp-2 indirect immunofluorescence assay (IIFA) is broadly used in order to detect ANA, and its outcome is included in both diagnostic and classification criteria when a systemic autoimmune disease is suspected. We agree with Damoiseaux J et al. that the HEp-2 IIFA test reveals a lot, considering the fluorescence pattern to be an important diagnostic tool which reveals clinically suitable information. It is important to reach a consensus in order to standardize the current diagnostic tools and achieve greater accuracy in our daily clinical practice, as well as aligning pattern descriptions across laboratories to homogenize the same criterion to help clinicians. We consider that the initiative by the International Consensus on ANA Patterns (ICAP) eases to be more accurate and emerge as an important tool in the diagnostic work-up, promoting the understanding of HEp-2 IIFA staining pattern nomenclature, as well as optimizing usage in patient care by providing new guidelines. Despite this, ANA test results still need to answer some questions, methodologic consensus statements and semantic issues.

Previous SjS reviews have focused on traditional autoantibodies, and then on some of the novel autoantibodies described in the recent years. Thus, there are two main works that analyze the usefulness of the different antibodies related to Sjögren’s syndrome. Firstly, Shen L et al. in 2017, discussed autoantibodies including the traditional ones, those identified initially from mouse models and those associated with other autoimmune diseases, examining their detection methods and their prevalence. Secondly, in 2019, Martín-Naresa E et al. mainly discussed their usefulness in order to identify a clinical subtype.

The advance in biological techniques for antigen determination have resulted in the knowledge of more auto-antigen specificities associated with autoimmune diseases, including SjS. Due to this heterogeneity in autoantibody responses and in clinical phenotypes, a broad-spectrum of diagnostic tools as antigen microarrays are increasingly demanded, targeting multiple specificities.

Conventional techniques used in the majority of diagnostic laboratories bring different non-specific initial screening methods based on the gold standard immunofluorescence assay of ANA autoantibodies or ELISAs, that use antigen mixtures. Although useful for its high sensitivity, these techniques would have to be evaluated for its efficacy in detecting patients with new autoantibody targets as positive. Some assays of ANA-ELISA have resolved this issue by adding a number of extra purified recombinant antigens, as is the case of some of the classical epitopes of Sjogren’s syndrome (Ro/SSA and La/SSB) and also in other rheumatic autoimmune diseases (98). To further identify the specificity of auto-antibodies, the initial screening is usually followed by a number of assays based on different technologies (i.e., line immunoassays, immunoblots or multiplex color-coded bead assays as Luminex^®^) that allow the study of only a reduced number of auto-antibody specificities. With the appearance of autoantibody arrays assays, the ability to measure a large number of autoantibodies with a small sample volume on a single platform matrix, opened the possibility of reducing the time and cost of performing several tests that included only small subsets of the most classical antigens. Thus, with the increasingly knowledge of novel auto-antibody targets, antigen microarrays may become an established tool for routine diagnostic procedures in the future. Also, these new technologies provide the opportunity of defining new autoantibody profiles and explore the relevance of different isotypes, also identifying patient subsets that could correlate with different prognostic outcomes or be candidates to novel personalized treatments. However, currently there are a number of obstacles when standardizing these new tools in daily clinical practice: issues in the design and optimization of all targets in a single array, the lack of available normalized standards protocols and the lack of batch-to-batch reproducibility and inter-laboratory comparison (99). On the other hand, given the importance of inflammatory cytokines in the pathogenesis of SjS, techniques with a more functional approach based on the measurement of cell expression of different immune-related molecules such as IFN, and its correlation with the presence of certain auto-antibodies, are being studied in other rheumatic autoimmune diseases with interesting results related to their possible role in the autoimmunity response in those patients (100).

Our work aims to combine the information collected in recent years regarding classic and novel autoantibodies in SjS, with a special emphasis on the role of ANA and the HEp-2 IIFA staining pattern, trying to define the usefulness of “organ-specific” biomarkers in the creation of new patient phenotypes that may step up the diagnosis of SjS in the future. In this way, as new biomarkers are identified, more subtypes of patients may be established, with their own intrinsic features, helping other physicians in the clinical suspicion of SjS and additionally guiding the clinician in the face of possible potentially serious complications. In this sense, we also emphasize the role of anti-Ro/SSA antibodies, review the antibodies shared with other autoimmune diseases and analyze the novel antibodies that could provide valuable information for understanding the pathophysiology of the disease, predicting new clinical profiles in the future and probably helping in the development of new therapeutic targets. Likewise, understanding and validating this new spectrum of autoantibodies may make it possible to carry out a diagnostic approach in those patients with negative anti-Ro/SSA antibodies with specific clinical phenotypes that do not meet the American College of Rheumatology/European League Against Rheumatism (ACR/EULAR) classification criteria.

To sum up, detecting serum antibodies is practical in order to determine both diagnosis and prognosis in autoimmune diseases. The identification of new autoantibodies in SjS opens up a window of opportunity to gain a better understanding of the SjS pathophysiology, to ascertain clinical phenotypes and to foresee long-term associated complications.

The characterization of autoantibodies and their target autoantigens in patients with primary SjS is helping to unravel more information regarding this common systemic autoimmune disease. Any positive serological result with clinically defined sicca symptoms or other extraglandular features, should raise red flags in the follow up of those patients. Some of the autoantibodies discussed in this review were identified in a research environment, but in the near future they may also be used as helpful tools in common clinical practice. Many of the autoantibodies mentioned in this review are found in subpopulations of SjS patients without any specificity and their real usefulness should be balanced and powered in later studies.

## Author contributions

All authors listed have made a substantial, direct, and intellectual contribution to the work and approved it for publication.

## Acknowledgments

We are grateful to Dr. David Cañadas Bustos for his comments that greatly improved the manuscript. We also thank to the autoimmunity section of the clinical laboratory of Hospital Universitario Gregorio Marañón for their help in the preparation of the images used in this review.

## Conflict of interest

The authors declare that the research was conducted in the absence of any commercial or financial relationships that could be construed as a potential conflict of interest.

## Publisher’s note

All claims expressed in this article are solely those of the authors and do not necessarily represent those of their affiliated organizations, or those of the publisher, the editors and the reviewers. Any product that may be evaluated in this article, or claim that may be made by its manufacturer, is not guaranteed or endorsed by the publisher.
